# Transitioning of protein substitutes in patients with phenylketonuria: evaluation of current practice

**DOI:** 10.1186/s13023-022-02555-8

**Published:** 2022-10-27

**Authors:** Ozlem Yilmaz, Alex Pinto, Anne Daly, Catherine Ashmore, Sharon Evans, Nurcan Yabanci Ayhan, Anita MacDonald

**Affiliations:** 1 Birmingham Women’s and Children’s NHS Foundation Trust, Birmingham, B4 6NH UK; 2 Department of Nutrition and Dietetics, Faculty of Health Sciences, Ankara Yildirim Beyazit University, Ankara, 06760 Turkey; 3 Department of Nutrition and Dietetics, Faculty of Health Sciences, Ankara University, Ankara, 06290 Turkey

**Keywords:** Phenylketonuria, Protein substitute, Transition, Barriers, Liquid, Powder

## Abstract

**Background:**

In children with phenylketonuria (PKU), transitioning protein substitutes at the appropriate developmental age is essential to help with their long-term acceptance and ease of administration. We assessed the parental experiences in transitioning from a second stage to third stage liquid or powdered protein substitute in patients with PKU.

**Results:**

Sixteen interviews (23 open-ended questions) were carried out with parents/caregivers of children with PKU (8 females, 50%) with a median age of 8 years (range 5–11 years), continuously treated with diet, and on a third stage protein substitute. Parents/caregivers identified common facilitators and barriers during the third stage protein substitute transition process. The main facilitators were: child and parent motivation, parent knowledge of the transition process, a role model with PKU, low volume and easy preparation of the third stage protein substitute (liquid/powder), anticipation of increasing child independence, lower parent workload, attractive packaging, better taste and smell, school and teacher support, dietetic plans and guidance, PKU social events, child educational materials and written resources. The main barriers were child aversion to new protein substitutes, poor child behaviour, child aged > 5 years, parental fear of change, the necessity for  parental time and persistence, loss of parental control, high product volume, different taste, smell, and texture of new protein substitutes, and peer bullying.

**Conclusion:**

A stepwise, supportive approach is necessary when transitioning from second to third stage protein substitutes in PKU. Future studies are needed to develop guidance to assist parents/caregivers, health professionals, and teachers during the transition process.

## Introduction

Protein substitutes are an integral part of dietary treatment in phenylketonuria (PKU) [1]. They allow the safe provision of all amino acids except phenylalanine and supply additional tyrosine that otherwise may be deficient. The amino acids provided by protein substitutes are essential for the synthesis of body protein and nitrogen containing compounds such as hormones, enzymes, and some neurotransmitters [1, 2]. Their overall composition and nutritional profile are fundamental: they prevent neurological damage, allow normal growth, and biosynthetic functions [2, 3].

There are different types of age specific protein substitutes, designed to meet the nutritional and developmental needs of children with PKU [4]. These include infant, weaning, child and adult formulae [1, 4]. Table 1 represents the general types and features of protein substitutes suitable for children below six years of age. Transitioning from one protein substitute to another at the appropriate developmental age is essential to establish acceptance, structure and routine into daily care, ease of administration, encouraging child independence with self-care, and to meet changing nutritional requirements with increasing age [1].

**Table 1:** Types of protein substitutes suitable for children < 6 years of age

Stage	Type	Features	Issues
1	Infant protein substitutes (powder/liquid) L-amino acids	• Phenylalanine-free • Given post neonatal diagnosis • Early infant exposure accustoms their taste to amino acids [4]	• Poor taste [4]
2	Semi-solid weaning protein substitutes L-amino acids	• Phenylalanine-free • Semi-solid consistency given from a spoon • Higher in protein equivalent than infant protein substitutes • Introduced from 6 months • Low volume/low energy so infant has capacity/appetite for solid foods [5, 6]	• Poor taste but most infants adapt if introduced at 6 months • Difficult to administer during teething/illness • Thickens on standing • High osmolality [5]
2/3	Powders suitable from 1y+ L-amino acids	• Phenylalanine-free • Concentrated in amino acids so low volume • Flexible as the amount of water added can be adjusted and it is usually given as a drink [4, 6]	• Poor taste • Less convenient • Needs water for preparation • High osmolality [7]
3	Ready to use liquid protein substitutes L-amino acids	• Phenylalanine-free • Low volume • Convenient • Usually low in carbohydrate, fat, and energy [7, 8]	• Poor taste • High osmolality [2, 4]
3	Casein glycomacropeptide with amino acids (CGMP-AA) Peptide based substitute with added amino acids	• Low-phenylalanine • Powdered, liquid and bars • Improved taste and palatability • May improve nitrogen retention • Prebiotic, antimicrobial and immunomodulatory effects [9–13]	• Contains residual phenylalanine • May increase blood phenylalanine in well-treated children if given as a sole source of protein substitute • Human studies on long-term effects are limited [1, 14–16]
3	Slow-release protein substitutes suitable from 3 + Amino acids coated with ethyl cellulose and alginate	• Phenylalanine-free • Granules • Mixed with food or fruit juice • Prolonged release and physiological absorption of amino acids shown in a non-PKU human study • Improved taste, smell and palatability [17, 18]	• Only short term studies reported [2, 19]

In early infancy, phenylalanine-free infant powders [4, 5] are reconstituted as liquids; they are delivered from an infant feeding bottle and mimic the nutritional profile of standard infant formula but are devoid of phenylalanine [4]. As infants gain weight, they need an increasing amount of infant protein substitute to meet protein requirements; but higher volumes may delay solid food progression by reducing appetite [20]. Therefore, from 6 months of age, gradual introduction of a second stage protein substitute with a higher protein equivalent but lower energy profile, administered in the form of a low volume, spoonable gel is common practice. It is easy to prepare with water, has a semi-solid consistency (similar to baby porridge), and contains age-appropriate vitamins and minerals [2, 5, 21]. Parents administer this type of protein substitute from a spoon.

The subsequent and third change in protein substitute type usually occurs between 3 and 5 years of age. This age is pragmatic and influenced by individual child maturation. In Northern Europe, the third stage protein substitute is usually a ready to use liquid or powder. It is more concentrated in protein equivalent, thus reducing the overall volume required but should be given with additional water [2, 7]. Changing protein substitutes at this childhood stage is challenging, and child resistance is common [22]. Thus, parents may defer this process, but any delay may lead to increased apprehension, anguish, and conflict for both parents/caregivers and child.

In PKU, some studies [5, 21, 23] have assessed the transition from phenylalanine-free infant formula to a second-stage, semi-solid protein substitute during weaning, but the transition from second to the third stage protein substitute is rarely described. In fact, there are no controlled studies that have reported the process of protein substitute transfer in children aged 3 years and over. This qualitative study aimed to assess the parental experiences in transitioning from semi-solid protein substitutes to age-appropriate ready-to-use liquid or powdered protein substitutes in children with PKU aged 3 to 11 years of age.

## Materials and methods

### Study design and settings

A qualitative open-ended questionnaire was designed in which one-to-one semi-structured interviews with parents/caregivers of children with PKU were performed. Their children had already transitioned from a second stage semi-solid weaning protein substitute to an age-appropriate third stage protein substitute.

Blood phenylalanine levels of children were collected for a median of 12 months before and after the protein substitute transition process to examine metabolic control of children. Prescribed total protein, natural protein, and protein equivalent intake from protein substitutes were calculated from dietetic records.

### Study population

Parents were recruited through purposive sampling from Birmingham Children’s Hospital, UK. Inclusion criteria were parent/caregiver of a child aged 3 to 11 years, diagnosed with PKU by newborn screening and continuously treated with diet following diagnosis, and fully or partially established on a third stage protein substitute. Children on sapropterin (BH4) treatment, with non-PKU comorbidities or with poor adherence to protein substitute were excluded.

**Fig. 1:**
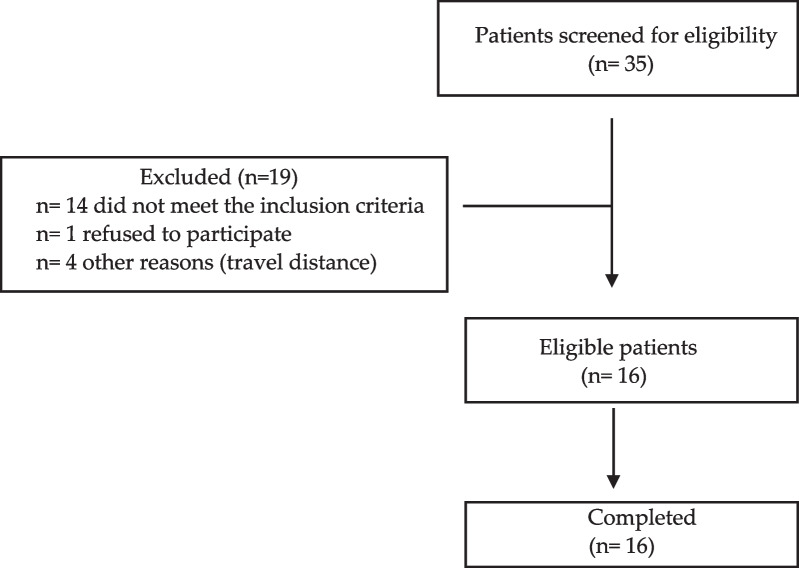
Flow chart of patients enrolled in the study

There were 35 children with PKU screened for eligibility. Nineteen children were excluded: Fourteen did not meet the inclusion criteria; 1 parent/child refused to take part and 4 children lived > 2 h travel distance from the hospital and could not be interviewed (Fig. 1).

### Procedure

Potential participants were identified based on the eligibility criteria. Recruitment was performed via telephone, home visits, or clinic visits after a patient information sheet had been posted to each parent/caregiver and they showed an interest in participating. They were then contacted via telephone to agree a date for an interview, and written informed consent was obtained before any data collection. Semi-structured interviews were conducted by one member of the research team (AM) with the main parent/caregiver of each child in the family home and it was audio-recorded with permission.

A total of 23 open-ended interview questions, covering the following themes were included: (1) a description of the length of time taken to transition of children from second to third stage protein substitute; including a description of parent approach to this process; (2) parental experiences during transition process; (3) anxiety of parents and children about protein substitute and the transition process; (4) parent/caregiver concerns; (5) enablers and challenges during the transition process; and (6) areas that required improvement or additional aid or support that may have facilitated this process. Participants were allowed to talk without restrictions or interruption about their experiences. Follow-up questions were asked if clarification was needed. A series of probes and prompts were used during the interviews. The interviews were approximately 60 min in duration and were transcribed verbatim. The audiotapes were destroyed once the information had been transcribed.

### Data analysis

Interview transcripts were analyzed using QSR NVivo11 qualitative analysis software (QSR International Pty Ltd., Melbourne, Australia) by the primary researcher (O.Y.). An inductive thematic analysis approach [24] was used to identify themes in the qualitative responses. Data analysis involved the six steps of thematic analysis described by Braun and Clarke [24]: becoming cognizant with the data; generating initial codes; searching for themes from the coded and collated text; reviewing coded text within each theme to add additional codes or themes; defining and further refining the themes; and conducting the final analysis with a final report of the findings with a set of worked-out themes. Based on the occurrence of redundancy after 16 interviews, the research team determined that data saturation was reached, and additional interviews were not required. Descriptive statistics were used to summarize the quantitative data.

### Ethics

This study was registered as an audit (CARMS-31022) based on guidelines from the National Health Research Ethics Service and it did not require ethical review. This was conducted in accordance with basic ethical principles, and participant confidentiality was maintained and in line with the Data Protection Act 2000. Written informed consent was obtained from parents/caregivers, and assent from children if age and understanding was appropriate.

## Results

### Participants

Sixteen semi-structured interviews were carried out with parents/caregivers of children with PKU (8 females, 8 males), who had been diagnosed by newborn screening. All had transitioned from a second stage semi-solid weaning protein substitute to an age-appropriate liquid or powder protein substitute. Ten interviews were with the mother only, and six were joint interviews with both mother and father. Demographic information and clinical characteristics of children are presented in Table 2. The median blood phenylalanine concentrations of children remained within the reference ranges of 120–360 µmol/L [4]: 12 months pre-protein substitute transition (median 215 µmol/L [range 110–340]), and 12 months post transition (median 240 µmol/L [range 130–390]).

**Table 2:** Demographic information and clinical characteristics of children with PKU

Variable	Number of children (n = 16)
Gender, n (%)	
Female	8 (50%)
Male	8 (50%)
Child age, median (range)	
At interview (years)	8 (5–11)
Birth order in the family, n (%)	
First and only child	5 (31)
First born	4 (25)
Second born	4 (25)
Third born	2 (13)
Fourth born	1 (6)
Blood phenylalanine levels in µmol/L, median (range)	
12 months before the transition	215 (110–340)
12 months after the transition	240 (130–390)
Prescribed total protein intake, median (range)	
Total protein (g/day)	65 (49.0–83.0)
Total protein (g/kg/day)	2.9 (1.8–3.6)
Natural protein (g/day)	5.0 (3.0–20.0)
Natural protein (g/kg/day)	0.2 (0.1–0.7)
Protein equivalent intake from PS (g/day)	60.0 (45.0–80.0)
Protein equivalent intake from PS (g/kg/day)	2.6 (1.7–3.3)

n = Number of patients; PS: protein substitute; g: gram; kg: kilogram

### Process of transitioning to third stage protein substitute

Table 3 describes the protein substitute transition process. All children (n = 16) were taking semi-solid second stage protein substitutes before transitioning onto the third stage protein substitute. The majority (n = 13, 81%) were transitioned to ready to drink liquid protein substitutes (e.g., PKU Cooler [Vitaflo International], PKU Air [Vitaflo International], PKU Lophlex LQ [Nutricia]). One child (6%) was transitioned to cGMP powdered third stage protein substitute (PKU Sphere [Vitaflo International]), and n = 2 (12%) a combination of a liquid and powdered third stage protein substitute (n = 1 to PKU Cooler + PKU Sphere, and n = 1 to PKU Air + PKU Express [Vitaflo International]). The choice of transition protein substitute was determined by the dietitian, dependent on its age suitability, nutritional profile, ease of administration, and accessibility. The child chose the flavour of the product they preferred.

**Table 3:** Third stage protein substitute transition history of children with PKU

Patient No	Gender	Type of second stage protein substitute	Type of third stage protein substitute	The age third stage protein substitute transition commenced (years)	The age third stage protein substitute transition finished (years)	Duration (months)
1	F	Semi-solid	Liquid	3.5	3.7	2
2	M	Semi-solid	Liquid	3.1	3.5	5
3	F	Semi-solid	Liquid	5.5	6.5	12
4	M	Semi-solid	Liquid	3.8	4.3	6
5	M	Semi-solid	Powder + Liquid^†^	5.2	6.6	17
6	M	Semi-solid	Liquid	5.3	5.3	0
7	F	Semi-solid	Powder	5.4	6.7	16
8	M	Semi-solid	Liquid	3.9	4.0	1
9	F	Semi-solid	Liquid	5.0	5.6	7
10	M	Semi-solid	Liquid	4.2	4.4	2
11	F	Semi-solid	Liquid	3.3	4.7	17
12	F	Semi-solid	Liquid	8.0	8.0	0
13	F	Semi-solid	Liquid	4.7	4.7	0
14	M	Semi-solid	Liquid	4.9	5.3	5
15	M	Semi-solid	Powder + Liquid^‡^	4.3	6.9	32
16	F	Semi-solid	Liquid	5.3	5.6	4

M: Male; F: Female

†Started transition onto cGMP powder and commenced on three doses of cGMP powder; the remainder were given as liquid amino acid protein substitute to ensure target blood phenylalanine levels were not exceeded

‡Started transition onto liquid, refused more than one dose of liquid so remaining doses were powdered protein substitutes concentrated in protein equivalent

The median age of commencement of the third stage protein substitute transition was 4.8 years (range 3.1–8.0 years). This process started from 3 to 4 years of age in 31% (n = 5/16), from 4 to 5 years in 25% (n = 4/16), from 5 to 6 years in 38% (n = 6), and at 8 years of age in 6% (n = 1) of children. The time of transitioning to third stage protein substitute was commonly associated with nursery or school commencement. The median age of children fully transitioning to third stage protein substitute was 5.3 years (range 3.5–8.0).

In 56% (n = 9/16) of children, the third stage liquid protein substitute was given in small volumes, gradually increased and offered in addition to usual protein substitute e.g., started at 10 ml/day and increased in 10 ml/daily increments at daily to weekly intervals until one full daily dose of protein substitute was established, and then it replaced a full dose of usual protein substitute. This was commonly conducted and supervised in nursery or school by teachers or teaching assistants. Of these children, the median duration of achieving one full dose of liquid protein substitute (around 90 ml) was 1 month (range 2 weeks to 4 months). In 13% (n = 2/16) of children, the third stage liquid protein substitute immediately replaced one full dose of daily protein substitute. One child (patient 12) was reluctant to try the third stage protein substitute until aged 8 years and then transitioned immediately to the full dose, encouraged by her younger sister with PKU who had previously transitioned her protein substitute.

In some children (n = 5/16, 31%), it took a substantial amount of time to fully transition to the full dose of third stage protein substitute (Table 3). One child (patient 7), who commenced on one daily dose of powdered third stage protein substitute at 5.4 years, fully transitioned to the prescribed dosage at 6.7 years of age. One patient (patient 15) transitioned to one daily dose of third stage liquid protein substitute at 4.3 years of age, refused more than one dose of liquid protein substitute, so the remaining doses were given as powdered third stage protein substitute, and by 6.9 years of age, he reached the full prescribed dosage of third stage protein substitute.

The metabolic control of children was satisfactory, with 85% of blood phenylalanine levels within the target range of 120–360 mol/L [1] with blood phenylalanine concentrations monitored weekly throughout the transition process. The median percentage of blood phenylalanine concentrations above the therapeutic target range of 120—360 µmol/L [1] were 13% in patients who took ≤ 2 months to transition (n = 6) and 13% in those who took > 2 months (n = 10). Any high blood phenylalanine levels were usually associated with infections. Only in one child (patient 8) who struggled to take his new liquid protein substitute could there have been a possible association between higher blood phenylalanine levels and lower adherence with the new protein substitute. This child took around 40 min to take each dose, but his protein substitute adherence improved with time.

Some parents (n = 4/16, 25%) reported fewer gastrointestinal problems and increased appetite after transitioning of their children to the third stage protein substitute. However, two parents (13%) reported poor tolerance such as gagging, and constipation related to third stage protein substitute.

### Qualitative findings

**Fig. 2:**
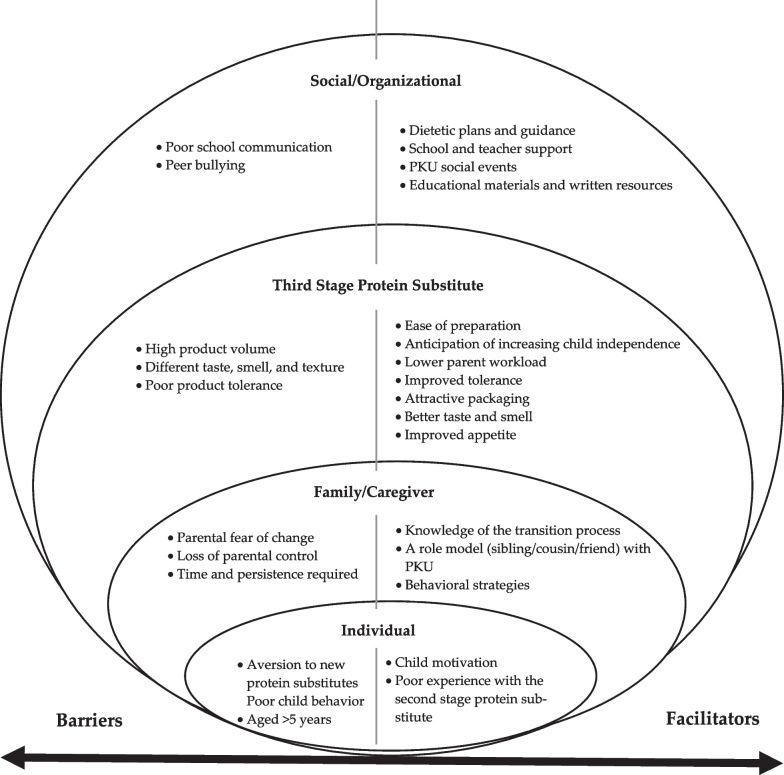
Perceived facilitators and barriers of the protein substitute transition process

Parents/caregivers reported a range of experiences about the protein substitute transition process and how this process could be improved. These findings were grouped into two broad categories: (1) facilitators and (2) barriers of the protein substitute transitioning process. Each category was subdivided into the following four groups of factors: (a) individual, (b) family/caregiver, (c) third stage protein substitute features, and (d) social/organizational. Figure 2 summarizes the perceived facilitators and barriers for each group. We also focused on parental suggestions on several strategies that could improve the protein substitute transition process.

#### Perceived facilitators during the transitioning to third stage protein substitute

##### Individual factors

Individual factors that successfully facilitated the protein substitute transition process included: (i) child motivation and (ii) poor experience with the second stage protein substitute. Some parents reported that their children showed signs of being willing to try third stage protein substitutes, and their desire for independence was an important motivator. ‘*So a trigger here was growing independence and it was going to hinder her going away independently if she didn’t start taking…’ (P12, transitioning age 8.0 years)*.

Children who struggled with the second stage protein substitute appeared willing to transition to the third stage protein substitute. *‘He was happy to try the -third stage protein substitute- because he didn’t like his -second stage protein substitute-.’ (P10, transitioning age 4.4 years).*

##### Family/caregiver factors

Family/caregiver factors that facilitated the protein substitute transition process included: (i) knowledge of the transition process, (ii) a role model (sibling/cousin/friend with PKU), and (iii) behavioral strategies. All parents/caregivers said they knew their child would eventually transition to third stage protein substitutes, which enabled them to prepare for the change. Many parents reported being given adequate information by their dietitian before the transitioning process. *‘We knew what was coming—we could prepare ourselves for a change but we did not have to start worrying about it too soon.’ (P4, transitioning age 4.3 years). ‘It’s something I learnt along the way because, like, our dietitian always mentioned it to us about once he goes to school he’ll have to start on changing to something more appropriate for his age.’ (P15, transitioning age 6.9 years).*

Some of the children were influenced by an older sibling or cousin who had gone through the same process. *‘I think it made it easier from our point of view because we’ve got two elder kids in the house with PKU…so he’d seen his brother and sister have it…so he knew that it was something that he had to have.’ (P8, transitioning age 4.0 years).*

Parents reported various behavioral strategies they used to facilitate the protein substitute transition process: encouragement and child rewards, a persistent and resolute approach, establishing a time routine and keeping a supply of the third stage protein substitute at home to get the child used to its appearance and taste from an early age. Parents often stated that encouraging their children by saying they were now a big boy or girl was helpful. Most parents reported offering rewards such as stickers, reward charts, or football cards to encourage the child for protein substitute change. Parents also used devices such as stopwatches and timers to help. Parents consistently emphasized that their children eventually adapted well to the change. *‘Yeah he got upset. Yeah he sort of resisted it. We just had to persevere with it…’ (P8, transitioning age 4.0 years)*. Parents reported that the slow transition and not forcing the child made the transition easier. They said that having a good routine helped so it became a regular part of a day. *‘We didn’t ever push him… any faster than he wanted to go… and I think, if we had done, he may well have pushed back and completely said no.’ (P2, transitioning age 3.5 years).* Some parents reported that keeping third stage protein substitute samples at home helped their children become familiar with the products. *‘Having samples at house did help a bit because we kept trying him with it.’(P15, transitioning age 6.9 years).*

##### Third stage protein substitute related factors

The third stage protein substitute related factors that facilitated the transition to a third stage protein substitute were: (i) ease of preparation, (ii) anticipation of increasing child independence, (iii) lower parent workload, (iv) improved tolerance, (v) attractive packaging, (vi) better taste and smell, and (vii) improved appetite.

Most parents reported that third stage liquid protein substitutes reduced parental workload, saved time, and were generally effortless to prepare. It was better when away from the home as there was no need to take scales, water, or spoons. *‘… it's easy when you are out and about that you can just, you know, shake it and open it …’ (P14, transitioning age 5.3 years).*

Some parents were satisfied because third stage protein substitutes gave more independence to their children and they were less socially isolated at school. *‘Giving her more independence really, especially when she’s at school… She can get involved and her friends actually think it’s really cool they think she’s having a milkshake. So you know, she can sit in the class and have it, she doesn’t feel…different.’ (P7, transitioning age 6.7 years).*

Parents also reported that the third stage protein substitute tasted and smelt better and had increased flavor options. Some parents also reported improved tolerance with fewer gastrointestinal problems and increased appetite. *‘I think one of the things that probably was a turning point with his liquid pouch was that he did start eating better. He has more of an appetite.’ (P6, transitioning age 5.3 years). ‘She’s got much less tummy ache on the third stage protein substitute.’ (P9, transitioning age 5.6 years).*

##### Social/organizational factors

Social and organizational factors that facilitated the protein substitute transition process included: (i) dietetic plans and guidance, (ii) school and teacher support, (iii) PKU social events, and (iv) educational materials and written resources.

All parents expressed satisfaction with the plans and guidance provided by the dietetic team during the transition process. Keeping to the instructions given by their dietitian was critical and reduced parental stress. ‘*We’ve always had great support. Yeah the dietitian was at the forefront of us changing.’ (P8, transitioning age 4.0 years).*

Most parents valued the importance of school/nursery support. Despite parental concerns about handing over responsibility to school/nursery teachers, most teachers played an active role in the transition process. *‘It was his teacher. She did most of the hard work really.’ (P14, transitioning age 5.3 years)*. *‘They were really good with that at nursery. They did time her. So, I would get a record of that as well from them every day.’ (P1, transitioning age 3.7 years).*

Observing other children while they took their protein substitute at PKU social events facilitated parents to prepare their children to transition. *‘It was a big thing seeing other older children taking the liquid pouches at the events… it really helped him.’ (P2, transitioning age 3.5 years).*

Some parents mentioned that having a written individual health care plan for parents/caregivers and the nursery facilitated the transition. *‘We were really clear from the start because we kind of had a structure of how we were going to do it from start to finish and we knew the timescales.’ (P1, transitioning age 3.7 years).*

#### Perceived barriers during the transitioning to third stage protein substitute

##### Individual factors

Individual factors that acted as barriers to protein substitute transition process included (i) aversion to new protein substitutes, (ii) poor child behaviour, and (iii) aged > 5 years.

Many parents reported their child's aversion to new protein substitutes and preference for the old protein substitute as the main barrier. *‘It was more of a struggle and she was reluctant to try it.’ (P12, transitioning age 8.0 years). ‘He has never been happy to change substitute. He is always a bit “oh no”. He sticks to what he knows normally.’ (P6, transitioning age 5.3 years).*

Parents described poor behaviors of their children as a barrier to the protein substitute transition process. These behaviors included: being stubborn or being quite definite in their views. *‘At the first start he was getting upset in that he wanted his old pouches back…and he was just “oh do I have to do this”.’ (P6, transitioning age 5.3 years).*

Parents of children who transitioned to third stage protein substitutes aged > 5 years reported experiencing more struggles. These children were often more attached to their second stage protein substitutes, resisted change, and needed effort and perseverance to change over to the third stage protein substitutes. *‘She got very attached to the second stage protein substitute so she got upset and refused to take the liquid for some time.’(P3, transitioning age 6.5 years).*

##### Family/caregiver factors

Family/caregiver factors that acted as barriers to the protein substitute transition process included (i) parental fear of change, (ii) loss of parental control, and (iii) time and persistence required.

Most parents felt anxious and worried about the transition due to the uncertainty of change. *‘I think any change makes you anxious, because it’s the unknown, and you have got control with what you have currently.’ (P2, transitioning age 3.5 years).*

Many parents were concerned about losing control over protein substitute administration because the third stage liquid protein substitute offers independence to the child. Parents are worried about their child’s capacity to take protein substitutes on their own. *‘The one thing that we did find most difficult was the fact that she was then independent in taking it. We had no control over it.’ (P1, transitioning age 3.7 years).*

Some parents reported feeling overwhelmed about the time and persistence required while incorporating the new protein substitute into their routine. *‘I think the thing that we found most hard was to stay positive as a parent …You need a lot of patience.It was a lot to be doing as well, the switching over and the remembering to do…’ (P1, transitioning age 3.7 years).*

##### Third stage protein substitute related factors

Third stage protein substitute related factors that acted as barriers during the transitioning process included (i) high product volume, (ii) different taste, smell and texture, and (iii) poor product tolerance.

Parents often reported that their child struggled to accept the prescribed volume of third-stage liquid protein substitute but also stated that their children became accustomed to the volume of liquid protein substitute over time, and it became less of an issue. *‘The volume was the problem and that’s what took us time.’ (P11, transitioning age 4.7 years).*

Although most children preferred the third stage protein substitute some children still struggled to take it due to the their different taste, smell, and texture. Parents frequently reported that their children were accustomed to the neutral taste of second stage protein substitute and found the flavoured taste of third stage protein substitute unpleasant. *‘It was introducing the taste because he was so used to the taste of the other product. He had taken this for ages and he is not adventurous with flavours. This was a big thing.’ (P4, transitioning age 4.3 years).*

Some parents reported poor tolerance such as gagging, gastrointestinal problems, and constipation related to third stage protein substitute. One parent observed that drinking third stage protein substitutes fast triggered the gagging reflex in their child. *‘He gags, he still gags now. If he drinks it too fast.’ (P15, transitioning age 6.9 years).*

##### Social/organizational factors

Social/organizational factors that acted as barriers to the protein substitute transition process included (i) poor school communication, and (ii) peer bullying.

Overall, more parents expressed positive experiences with the school, but there were a small number of parents expressing dissatisfaction with the school's lack of feedback. *‘The feedback mechanism is important. Yes that’s where it is massively lacking with the school.’(P16, transitioning age 5.6 years).*

Some parents identified child isolation or peer bullying as a particular concern. ‘*Kids are laughing in school. They don’t understand what he is taking… and we don’t want kids laughing at him because of whatever he is drinking.’ (P5, transitioning age 6.6 years).*

#### Parental suggestions to improve transitioning process

Parents gave several ideas that would improve the transition process. Many said that a child storybook with pictures explaining the protein substitute transition process would be beneficial. Some parents suggested creating storybooks with characters taking protein substitutes in different settings such as in school or on holidays. *‘… I think having a book aimed at their age group explaining what it is and that it's, you know, a big boy drink or a big girl drink… and they're gonna have to move on to it, I think that would be very good.’ (P14, transitioning age 5.3 years).* Some parents stated seeing videos and short videoclips of children drinking protein substitutes would be helpful. *‘All I can think is some recorded video which you can watch kids taking it.’ (P5, transitioning age 6.6 years).*

Some parents requested written instructions given to the school to facilitate the protein substitute transition process with formal feedback that would be helpful. *‘We expect teachers to report back on a daily basis.’ (P16, transitioning age 5.6 years).*

Mothers were primarily responsible for the dietary treatment of their children, and they expressed their desire for more involvement of other family members e.g. fathers and grandparents, to share the workload. *‘Don’t always be the one to give it, let other people help you. So the pressure is not always on you.’ (P11, transitioning age 4.7 years).*

Perceptions of parents on the variety of the third stage protein substitutes available differed. Some of them reported a good variety and suggested more options would cause difficulties. *‘I think it is better not to have too much choice sometimes. I think it would have caused confusion.’ (P2, transitioning age 3.5 years).* However, some parents expressed their wish to have more third-stage protein substitute options with different textures and flavours. *‘I would have liked more choices for my son to try’ (P10, transitioning age 4.4 years), ‘..it would have helped if we had more choices in flavours and textures.’ (P3, transitioning age 6.5 years).*

## Discussion

This qualitative study is the first to identify multiple facilitators and barriers experienced by parents of young children with PKU during the transition from a semi-solid (second stage) protein substitute to age-appropriate liquid or powdered (third stage) protein substitutes. The median age of commencing the transition process with the third stage protein substitute was 4.8 years (range 3.1–8.0 years). Later introduction of third stage protein substitute (> 5 years of age) appeared to result in more refusal or resistance to change. Parents encountered several barriers during the protein substitute transition process and acknowledged the importance of persistence and an unwavering approach with consistent support by peers, other family members, the PKU specialist dietitian, and school/nursery teachers. Parents also suggested several strategies such as storybooks, videos, clips, and written instructions for schools and nurseries that could improve the transition process.

Administration of protein substitute remains a significant time and emotional burden for parents [25] and it is essential to make this process more comfortable for the child and parents. Parents have previously described the daily labours of administering the protein substitute at least three times every day, describing high levels of stress and anxiety and have even said that this destroys their family life [25–30]. In our study, parents expressed their concern about the uncertainty of change and losing control over protein substitute administration knowing that the third stage liquid protein substitute would lead to child self-responsibility and autonomy. Some parents had continued to spoon feed the semi-solid protein substitute to their child beyond the age of 3 years to ensure they consumed it. From the interviews, it was evident that parenting styles, environment and anxiety influenced the success or breakdown of the transition process. A positive and calm parental style was the key to success. Important elements identified by parents that aided the process were: consistent and reassuring dietetic mentoring support, gaining school/teacher support to assist with the transition process, and discussion with other parents of PKU children who had been through this process that provided practical and emotional support.

It is already established that many young children with PKU are food neophobic and find change particularly difficult [22, 31]. Studies show that children with PKU are more particular about what they eat, consume less variety than non-PKU children and are less trusting and more fearful of new foods [22, 30]. The majority of the children included in this study were content with their second stage protein substitutes and initially resisted any transition of protein substitute. However, later introduction of third stage protein substitute (age > 5 years) appeared to be associated with more refusal or resistance. Most children adapted to the change with a slow, gradual but consistent introduction of a new, unfamiliar protein substitute. Parents described several behavioural strategies they used to facilitate the transition process e.g. encouragement and small rewards, tenacious and an unwavering parental approach with good routine, enabling the child to become accustomated to the third stage protein substitute by keeping a small supply at home prior to its introduction and talking to children about its introduction. They confirmed that following the stepwise guidance provided by a specialist dietitian was beneficial.

Most children (n = 13/16) in our study cohort were the firstborn with PKU in a family. In children with an older sibling with PKU, the median duration of transition to the third stage protein substitute was lower (1 month [range 0–2 months]) compared to firstborn children (6 months [range 0–32 months]) with PKU. In addition, children with an older sibling with PKU fully transitioned to third-stage protein substitutes around eight months earlier (median age: 4.2 years [range 3.9–4.7 years]) than the firstborn PKU children (median age: 5.0 years [range 3.1–8 years]). Older siblings may serve as role models and provide extra encouragement to the younger child with PKU. Parents understood the challenges of the transition process better, and they also knew that they would move through this process, even if they met obstacles along the way. However, poor previous experience of transition may cause reluctance to change protein substitute type, leading to overall delay in the process.

Our results also suggested that generally the third stage protein substitute improved tolerance with fewer gastrointestinal problems and improved child appetite but this was not consistent in all children. Second stage protein substitutes designed for infants from weaning age contain starch which may decrease gastric emptying time and thus suppress appetite [20, 32]. They are also hyperosmolar, and if given with minimal additional water, may cause abdominal pain, diarrhoea or constipation. It is recommended that additional water is given in concurrence with administration of all protein substitutes [4] but this is commonly omitted in practice.

In PKU, protein substitute adherence is an ongoing issue [1, 4, 33–35]. Recent evidence suggests that innovations in its taste, volume, presentation and improved access by home delivery has improved long-term adherence, particularly in teenagers taking liquid protein substitutes [7, 8, 36]. In our study, most parents reported that the third stage protein substitute tasted and smelt better and had increased flavour options, although their children did not necessarily appreciate or comprehend these advantages. Ready to use liquid protein substitutes reduce parental workload, save time and are packaged in pouches that are not readily recognisable as a medical food. However, some of the children had difficulties in their acceptance usually associated with the higher volume or different flavour and taste. Although this became less of an issue with age, it is important that children have a range of suitable age-appropriate protein substitute options to choose from. Transitioning of protein substitute at the appropriate age may lead to better long-term adherence and acceptance of protein substitutes.

Parents expressed concern about child isolation or peer bullying in school. This is in line with reports from other recent studies [25, 37] showing that the perception of social isolation and the dietary stigma are obstacles for successful PKU management. Perceived stigmatization is associated with worse psychological health [25, 38], and so patients need to be supported with suitable strategies [39] such as social skills training or school-based interventions in which peers are provided with basic information about the condition, resulting in better understanding [40]. In our study, some children had their own role models/peers with PKU who gave them confidence that they could also cope in social situations when they took their protein substitute publicly. Some parents stated that the attractive packaging of third-stage protein substitutes reduced the social stigma at school, and a mother described how her daughters friends described the liquid protein substitute pouch as ‘cool’ which increased her self-esteem.

## Limitations

This is a single-centre study with a small number of participants, but it is likely that our findings are relevant to PKU populations in other countries. The children’s own dietitian conducted the interview, so this may have tempered some of the answers given, but all parents were pleased to have the opportunity to give an open, candid and detailed account. The parents were asked the same questions and participants were given plenty of opportunity and time to talk in a relaxed, home environment. We did not collect data about parents coping ability, parental anxiety or stress or formally assess child’s behaviour.

## Conclusion

The findings of the present study highlight the importance of the slow and gradual introduction of a third-stage protein substitute with consistent support given by the PKU team. Although parents in our study had received intensive dietetic support, the parental time, persistance and patience required for the successful transition of third stage protein substitute was high. The involvement of other family members, school and teacher support, attending PKU social events, and encouraging children with several behavioral strategies provided practical and emotional support. Starting the transition of protein substitutes between 3 and 5 years of age appeared important and may aid successful long term adherence and acceptance. Future controlled, longitudinal studies are needed to develop practical and useful resources for parents, nursery and health care professionals to facilitate the protein substitute transition process and identify the ideal age for this process to begin.

## Data Availability

The data presented in this study are available on request from the corresponding author**.**

## Abbreviation

PKU: Phenylketonuria
